# The carboxyl termini of RAN translated GGGGCC nucleotide repeat expansions modulate toxicity in models of ALS/FTD

**DOI:** 10.1186/s40478-020-01002-8

**Published:** 2020-08-04

**Authors:** Fang He, Brittany N. Flores, Amy Krans, Michelle Frazer, Sam Natla, Sarjina Niraula, Olamide Adefioye, Sami J. Barmada, Peter K. Todd

**Affiliations:** 1 Department of Neurology, University of Michigan, 4005 BSRB, 109 Zina Pitcher Place, Ann Arbor, MI 48109-2200 USA; 2 Department of Biological and Health Sciences, Texas A&M University, 700 University Blvd, Kingsville, TX 78363 USA; 3 Neuroscience Graduate Program, University of California at Los Angeles, Los Angeles, CA USA; 4 Veterans Affairs Medical Center, 4005 BSRB, 109 Zina Pitcher Place, Ann Arbor, MI 48109-2200 USA

**Keywords:** *C9ORF72*, Amyotrophic lateral sclerosis, Dipeptide repeat proteins (DPRs), Repeat-associated non-AUG translation (RAN), Carboxyl terminus, *Drosophila*

## Abstract

An intronic hexanucleotide repeat expansion in *C9ORF72* causes familial and sporadic amyotrophic lateral sclerosis (ALS) and frontotemporal dementia (FTD). This repeat is thought to elicit toxicity through RNA mediated protein sequestration and repeat-associated non-AUG (RAN) translation of dipeptide repeat proteins (DPRs). We generated a series of transgenic *Drosophila* models expressing GGGGCC (G_4_C_2_) repeats either inside of an artificial intron within a GFP reporter or within the 5′ untranslated region (UTR) of GFP placed in different downstream reading frames. Expression of 484 intronic repeats elicited minimal alterations in eye morphology, viability, longevity, or larval crawling but did trigger RNA foci formation, consistent with prior reports. In contrast, insertion of repeats into the 5′ UTR elicited differential toxicity that was dependent on the reading frame of GFP relative to the repeat. Greater toxicity correlated with a short and unstructured carboxyl terminus (C-terminus) in the glycine-arginine (GR) RAN protein reading frame. This change in C-terminal sequence triggered nuclear accumulation of all three RAN DPRs. A similar differential toxicity and dependence on the GR C-terminus was observed when repeats were expressed in rodent neurons. The presence of the native C-termini across all three reading frames was partly protective. Taken together, these findings suggest that C-terminal sequences outside of the repeat region may alter the behavior and toxicity of dipeptide repeat proteins derived from GGGGCC repeats.

## Introduction

Nucleotide repeat expansions are a common cause of neurodegenerative disease. Transcribed repetitive sequences can elicit toxicity through at least two possible mechanisms. First, repeats as RNA can bind to and sequester specific proteins and preclude them from performing their normal functions [1]. Alternatively, repeats can be translated into proteins that accumulate and elicit toxicity in model systems [2]. Recently, it was recognized that translation of these repetitive sequences can occur in the absence of an AUG start codon through a process known as repeat-associated non-AUG (RAN) translation [3, 4]. As such, repeat expansions located outside of protein coding open reading frames can also produce toxic proteins.

In 2011, two groups simultaneously identified GGGGCC repeat expansions as a cause of amyotrophic lateral sclerosis and frontotemporal dementia (C9ALS/FTD) [5, 6]. This mutation is common, explaining upwards of 40% of all familial cases of ALS and FTD and 10% of all sporadic cases of these conditions. Most people have only a few repeats, with a cutoff for normal defined as less than 22–28 [7]. Pathologic expansions are usually hundreds to thousands of repeats. The repeat resides in the first intron of *C9ORF72*, a gene that is highly expressed in the brain but whose function remains unclear, though evidences indicate the encoded protein may act as guanine exchange factors for activating Rab proteins [8–10]. It is bi-directionally transcribed, such that both G_4_C_2_ and C_4_G_2_ repeat RNAs are produced, and RNA foci from both transcripts are observed in patient-derived cells and tissues [11–13]. Moreover, products of RAN translation are produced from both of these transcripts, leading to 6 different dipeptide repeat (DPR) containing proteins [11, 12, 14, 15]. Repeat expansions also impact *C9ORF72* transcription, isoform choice, and splicing [5, 14, 16–18].

A series of model systems have provided insights into C9ALS/FTD pathogenesis. *Drosophila* models demonstrate repeat-associated toxicity when the repeat is placed within the 5′ untranslated region (UTR; hereafter referred to as 5′ leader) of a transgene such as GFP [19]. In contrast, long repeats interrupted by stop codons elicit little toxicity [20, 21]. Flies expressing dipeptide repeat proteins via AUG initiated translation and independent of G_4_C_2_ repeat sequences are also toxic when expressed in flies and cells in some, but not all reading frames. Specifically, toxicity appears greatest with expression of glycine-arginine and proline-arginine repeat proteins in *Drosophila,* with evidence for a role of glycine-alanine proteins in mammalian neurons and model systems [20–25]. Mouse models using interventricular adenoviral delivery of 66 or 149 G_4_C_2_ repeats in isolation exhibit RNA foci, RAN translation, neurodegeneration, and motor phenotypes [26]. Taken together, these studies do not preclude a role for the repeat RNA in disease toxicity, but suggest that RAN translation directly contributes to neurodegeneration in C9ALS/FTD.

Less attention has thus far been placed on the native sequence context of the repeat. The initial screening of expansion of G_4_C_2_ repeats near ALS loci found that though such repeats are quite common in human genome, the expansion of G_4_C_2_ repeats were only detectable in *C9ORF72* gene but not in other ALS loci genes [27], indicating that the context of the expanded repeats at *C9ORF72* gene are quite unique for expanded repeat toxicity. The sequence context may well be critical to determining the final location of the repeat RNA, its interactions with translational machinery, and the RNA binding proteins with which it interfaces. Tran et al. addressed this issue in *Drosophila* by providing evidence that (a) repeats located within introns are less toxic than repeats placed in 5’m^7^G capped and polyadenylated transcripts, and (b) this enhanced toxicity was associated with increased DPR production [21, 28]. However, sequence context may also alter the final translated peptides produced by RAN translation and this could influence their toxicity. For example, in Huntington disease, expression of a *huntingtin* (*HTT*) isoform containing exon 1 alone with the CAG repeat produces a highly toxic and aggregate-prone factor that accumulates in patient brains [29, 30]. More recently, Sellier et al. demonstrated a role for the carboxyl terminus (C-terminus) of FMRpolyG, a RAN translated protein derived from a CGG repeat expansion in the Fragile X gene, as critical for repeat toxicity in models of Fragile X-associated Tremor Ataxia Syndrome (FXTAS) [31]. We therefore sought to explore whether the repeat location within a transcript and the sequences 3′ to the repeat that might influence the final RAN translated products, therefore influence the GGGGCC repeat toxicity in disease models.

Here we show that expression of up to 484 interrupted G_4_C_2_ repeats within a *Drosophila* intron exhibit minimal toxicity, largely consistent with prior studies [20, 21, 28]. In contrast, relatively short (28) repeats placed in the 5′ UTR of GFP elicited marked toxicity in *Drosophila* in some, but not all, reading frames relative to a downstream reporter. This differential toxicity correlated with the size and content of the C-terminus of the GR reading frame: with larger C-termini or GFP fusions leading to low toxicity. The relative toxicity observed in these *Drosophila* models correlates with nuclear accumulation of DPR-containing proteins and is recapitulated in rodent neurons. The inclusion of the native C-termini provides some neuroprotective effects. Taken together, these findings suggest that repeat context has a significant influence on its toxicity and identifies a new role for the C-termini in modulating DPR toxicity across model systems.

## Materials and methods

### Construction of mammalian transfection vectors

Constructs for mammalian expression experiments were generated by digesting GFP (G_4_C_2_)_71_ pcDNA3.2 (a gift from Christopher Shaw) with XbaI and inserting the resulting (G_4_C_2_)_71_ containing fragment into pcDNA3.1 containing GFP. One to 2 nucleotides were inserted upstream of GFP to result in each reading frame. Using NheI and PmeI restriction enzymes, the (G_4_C_2_)_71_ GFP fragment was inserted into pGW for neuronal expression. To generate plasmids with or without the native C-termini, a small fragment was inserted in place of GFP using AscI and MfeI in pGW. The ATG-V5 fragments were inserted upstream of the repeat using Acc65I and NotI in pGW.

### *Drosophila* stocks and genetics

All flies were maintained with standard food and culture conditions at 25 °C, while all crosses were made and maintained at 29 °C unless otherwise stated. Fly lines acquired from the Bloomington Stock Center were: GMR-GAL4 (#8605), and UAS-GFP (multiple lines). Other lines used were the ubiquitous driver Actin5C-GAL4/CyO (a gift from Zhe Han), the motor neuron specific driver OK6-GAL4 (a gift from Cathy Collins), and a RU-486 inducible Geneswitch Tubulin-GAL4 (Tub5) driver line (gift from Scott Pletcher).

The sequences for intronic and 5′ leader fly lines are detailed in Supplemental Table S1. Briefly, for intronic repeat lines, a mini intron from the fly *Prospero* gene [32] was inserted into the middle of GFP, synthesized commercially (Genewiz, NJ), and cloned into the EcoRI-XbaI site of pUAST. An intronic fragment with either 3 or 28 G_4_C_2_ repeats along with 40 nt of upstream and 260 nt of downstream intronic sequence from human *C9ORF72* was PCR amplified according to [33] from genomic DNA and inserted into the intron. The 21 repeat tiling fragment was generated using primer NotI-C9 AnchorR+PspOMI tiling (Supplemental Table S2). The 28 repeat insert was generated using primer NotI-C9R + XhoI-C9F directly from genomic DNA of an ALS patient with 28 repeats. Repeat blocks of (G_4_C_2_)_21_ bracketed by NotI and PspOMI restriction sites were then serially inserted next to this repeat, producing concatamerized intermediate 49, 70, 91, and 121 repeat containing constructs (Table S1). This element was then further concatamerized to generate lines with 242 (GFP-iC_242_) or 484 (GFP-iC_484_) interrupted repeats.

5′ leader repeat lines were generated by cloning the same 28 repeat sequence described above with approximately 30 nt on either side of intronic sequence into the 5’UTR of pEGFP-N1 (Takara Bio USA, CA) in the 0+ reading frame. The repeat and GFP were then sub cloned into the NotI site in pUAST. To induce frameshifts, annealing primers were inserted into the AgeI site between the repeat and the AUG of GFP (Supplemental Table S2). pUAST vectors carrying the respective inserts were used to generate transgenic lines by standard p-element insertion (BestGene, CA).

For the fly lines with or without native C-termini, the sequence flanking the (G_4_C_2_)_69_ repeats in constructs for neuron transfection were digested by KpnI and XbaI, and the inserts were cloned to the vector pUAST-AttB (Drosophila Genomics Resource Center, IN) digested with the same set of enzymes. The resulting pUAST-AttB vectors were then used for site-specific transgenesis using PhiC31 integrase technique to locus AttP40 [34] (BestGene, CA). All constructs were verified by Sanger sequencing and agarose gel sizing (Supplemental Figure S1A).

### Eye phenotype imaging and quantification

Representative fly eye images were taken by Leica M125 stereomicroscope and photographed with a Leica DFC425 digital camera as previously described [35]. Eye morphology of 1-2d post eclosion flies was quantitatively scored as previously described [36]. Briefly, we used the following criteria: supernumerary inter-ommatidial bristles, abnormal bristle orientation, ommatidium fusion, ommatidium pitting, disorganization of ommatidial array, and retinal collapse. The presence of each feature was given 1 point. An additional 2 points were given if more than 5% of the eyes were affected or 4 points if more than 50% of eyes were affected. Higher scores mean the eyes were more degenerated. Over 100 flies were scored per genotype in at minimum of three independent crosses. Scores were calculated and are presented as mean ± SEM.

### Fly viability and eclosion rates

Analysis of eclosion rates were performed as described [37]. Each transgenic line was crossed to the ubiquitous driver Actin5C-GAL4 (act5C-GAL4) balanced over a marker chromosome (CyO), on standard food at 29 °C. If the transgene elicited no toxicity, then 50% of progeny should have the CyO marker and 50% express the transgene. Over 100 flies of each genotype were scored over multiple crosses. The relative percent progeny carrying the transgene were expressed as a percent of total eclosed flies. Analysis of viability post eclosion was performed as described [38].

### Larval crawling assay

Transgenic lines were crossed to the motor neuron specific driver line OK6-GAL4 at 29 °C and the 3rd instar larvae with desired genotypes were collected. The crawling assay was performed with slight modification from [39]: 3rd instar larvae were collected by adding 20% sucrose on the food, briefly washed with MilliQ water, and then maintained in a water droplet until being analyzed. The larvae were accommodated at room temperature on 2% agarose gel in 10 cm petri dish for 1 min, and then the crawling distance in the next 1 min was recorded and processed by ImageJ. At least 50 larvae from the appropriate genotype were recorded and pooled as the same genotype.

### *Drosophila* lifespan assay

The UAS transgenic lines were crossed to Tub5-GAL4 (Tub5) Geneswitch driver flies on standard food absent of RU-486 at 29 °C. Adult offspring of the desired genotypes were collected 2-3d after eclosion and transferred to standard fly food containing 200 μM RU-486 without yeast granules. The flies were transferred to fresh food with drug every 3-4d. Each genotype started with at least 4 vials of 25 flies/vial (2 vials of males and 2 vials of females) and the survival was determined daily or every other day for 50d or until all flies were dead. For each genotype, at least 2 individual lines were examined.

### Generation of specific dipeptide antibodies

The rabbit polyclonal antibodies were generated by Abclonal (Cambridge, MA). Synthetic peptides corresponding to the repeat region containing 6 repeats were generated. All antibodies were affinity purified from anti-sera prior to use. Characterization of GA and GR specific antibodies was performed as previously described [40]. Characterization of the GP specific antibody can be seen in Supplemental Figure S2.

### Western blotting

Protein quantification was performed as previously described and imaged on standard film [41, 42]. Briefly, fly head or cell lysates were lysed in RIPA buffer with protease inhibitors (Sigma Aldrich, MO) and passed through a 27-gauge needle to shear DNA. Equal amounts of protein were run on a 12% SDS polyacrylamide gel. After transfer to PVDF membrane, blots were incubated with the following antibodies: monoclonal mouse anti-GFP (Sigma Aldrich, MO; clone 7.1 and 13.1, 1:1000), mouse anti-Tubulin (DSHB, IA; clone E7, 1:1000), mouse anti-V5 (Thermo Fisher, CA; 1:1000), rabbit anti-GA (Abclonal, MA 1:100), rabbit anti-GP (Abclonal, MA 1:5000), rabbit anti-GR (Abclonal, MA; 1:5000) or mouse anti-β-Actin (Sigma Aldrich, MO; 1:5000). At least three independent experiments were performed and scanned films were processed and quantified using ImageJ software.

### In situ hybridization

1-3d post eclosion fly heads from each genotype crossed to GMR-GAL4 driver were isolated, immediately frozen in OCT media, and then cryosectioned to 10 μm. Transverse sections were then fixed with 4% paraformaldehyde in 1x PBS for 15 min, washed 3x in 1x PBS, and permeabilized with 2% acetone for 5 min. In situ hybridization was performed as follows: sections were pre-hybridized with 50% formamide in 2x SSC for 30 min at room temperature and then hybridized with 500 μL hybridization solution in a light proof box for overnight at 37 °C. The hybridization solution was as follows: 0.6 ng/mL Cy5 labeled 2′-O-Me-(CCCCGG)_5_ RNA probe (IDT DNA Technologies, IA), 0.02% BSA, 132 mg/mL yeast RNA (Sigma Aldrich, MO), 1 μL RNAse inhibitor (Sigma Aldrich, MO), 50% formamide in 2x SSC. After hybridization, slides were washed with 3 × 30 min with 50% formamide in 0.5x SSC at 56 °C, followed by 3x10min washes with 0.5x SSC wash at room temperature. After washing, samples were dried for 5 min, incubated with 100 μL Prolong Gold with DAPI (Thermo Fisher, MA) for 1 h, and examined on an Olympus FV1000 confocal microscope with identical laser settings for each slide. Images were overlaid and quantified using ImageJ software.

### Fly based immunofluorescence

1-3d post eclosion fly heads from each genotype crossed to GMR-GAL4 were isolated, immediately frozen in OCT media, and then cryosectioned to 10 μm. Transverse sections were then fixed with 4% paraformaldehyde in 1x PBS for 15 min, washed 3x5min with 1x PBS, and permeabilized with 0.1% triton X-100 in 1x PBS for 5 min. Slides were pre-incubated with 5% normal goat serum in 1XPBS for 1 h at 4 °C, then incubated with antibodies of GA (1:100), GP (1:500), or GR (1:500) diluted in 5% normal goat serum in 1XPBS at 4 °C overnight. Slides were washed 2 × 5 min with 1x PBS. Slides were then incubated with Alexa fluo-568 goat anti-rabbit secondary antibody (Abcam, Cambridge, MA) diluted in 5% normal goat serum in 1x PBS for 30 min at room temperature in a dark box. After 3 × 5 min washes, slides were dried 5 min and then incubated with 100 μL prolong gold with DAPI (Thermal Fisher, MA) for 1 h and then were sealed with nail polish. Slides were examined with Olympus FV1000 confocal microscope with identical laser setting for each slide. Images were overlaid in ImageJ software, and 1 random-selected region of interest (ROI) in retina area of one fly head was analyzed and at least 10 fly heads were analyzed for each genotype.

### Cell based immunocytochemistry

COS-7 cells were maintained 37 °C in 5% CO_2_ incubators. Dulbecco’s modified Eagle’s medium (DMEM) supplemented with 10% fetal bovine serum and 1% Pen-Strep was used as culture media. For immunofluorescent detection of GFP, cells were cultured on 4 well chamber slides. Cells were transfected using Lipofectamine LTX with Plus Reagent (Thermo Fisher, MA) using manufacturer’s protocol. 48 h after transfection, cells were fixed with 4% paraformaldehyde for 15 min, washed with 1x PBS, and permeabilized 0.1% triton X-100. Cells were then blocked with 5% normal goat serum in 1x PBS containing 0.1% triton X-100 for 1 h and incubated with GFP (1:1000) plus GA (1:100), GP (1:500), or GR (1:500) antibodies overnight at 4 °C. Slides were washed with 1x PBS and incubated with AlexaFluor 488 labeled goat anti-mouse (Thermo Fisher, MA; 1:500) and Alexa Fluor 555 labeled goat anti-rabbit (Thermo Fisher, MA; 1:500) antibodies and visualized with an Olympus epifluorescence microscope with Slidebook 5.5 software with identical fluorescent settings for each slide.

### RNA isolation and qRT-PCR

Total RNA was extracted from 15 fly heads of each genotype using Trizol (ThermoFisher, MA) and quantified by Nano-Drop (Thermo Fisher, MA). 1 μg of total RNA was then reverse-transcribed to cDNA by iScript cDNA synthesis kit (Bio-Rad, CA). The primers used are detailed in Supplemental Table S2. PCR analysis was performed using iQ SYBR Green Supermix on a MyiQ Single Color qPCR system (BioRad, CA). All runs included a standard dilution curve representing 2x to 0.02x of the RNA concentration utilized for all primer sets to ensure linearity. Equivalent efficiency of individual primer sets was confirmed prior to data analysis. The level of *GFP* was normalized to *RPL32* mRNA for each sample run and expressed as a ratio of levels to GFP-iC3 lines (fold control expression) unless otherwise stated. All samples were run in triplicate in each qPCR run and all data represent at least three independent experiments.

### Primary neuron cultures & transfection

All mammalian rodent work was approved by the Institutional Animal Care and Use Committee (IACUC) at the University of Michigan. Primary mixed cortical neurons were dissected from embryonic day 19–20 Long-Evans rat pups and cultured at 6.0 × 10^5^ cells/mL in 96 well cell culture plates as previously described [43–45]. Brains of a single litter were combined to maximize cell counts, resulting in neurons from a mixed population of male and female pups. Neurons were cultured in NEUMO photostable medium containing SOS supplement (Cell Guidance Systems, MO) at 37 °C in 5% CO_2_. After 4d in culture, neurons were transfected with 0.2 μg DNA (total) and 0.5 μL Lipofectamine 2000 (Thermo Fisher, CA) per well in a 96 well plate, as described previously [45]. Neurons were incubated with the DNA/Lipofectamine 2000 mix for 20 min at 37 °C before rinsing. The remainder of the protocol followed the manufacturer’s protocol.

### Longitudinal fluorescence microscopy

Automated longitudinal fluorescence microscopy began 24 h post-transfection for 10d, as previously described [43–45]. Images were acquired using an inverted microscope (Nikon Instruments, NY) with a 20x objective lens, a PerfectFocus system, a Lambda XL Xenon lamp (Sutter Instruments, CA) with 5 mm liquid light guide (Sutter Instruments, CA), and either an Andor iXon3 897 EMCCD camera or Andor Zyla4.2 (+) sCMOS camera. All stage, shutter, and filter wheel movements were done using a custom code written in μManager, ImageJ [45].

### Statistical analysis

For most fly comparisons, scores were analyzed using a non-parametric Kruskal-Wallis ANOVA with Dunn’s correction for multiple comparisons. A Log-rank (Mantel-Cox) Chi square test was performed for *Drosophila* survival rates. Error bars represent the standard error of the mean except for proportion numbers, where the error bars represent the 95% confidence interval.

## Results

### Generation of an intronic repeat model of C9ALS/FTD in *Drosophila*

**Fig. 1:**
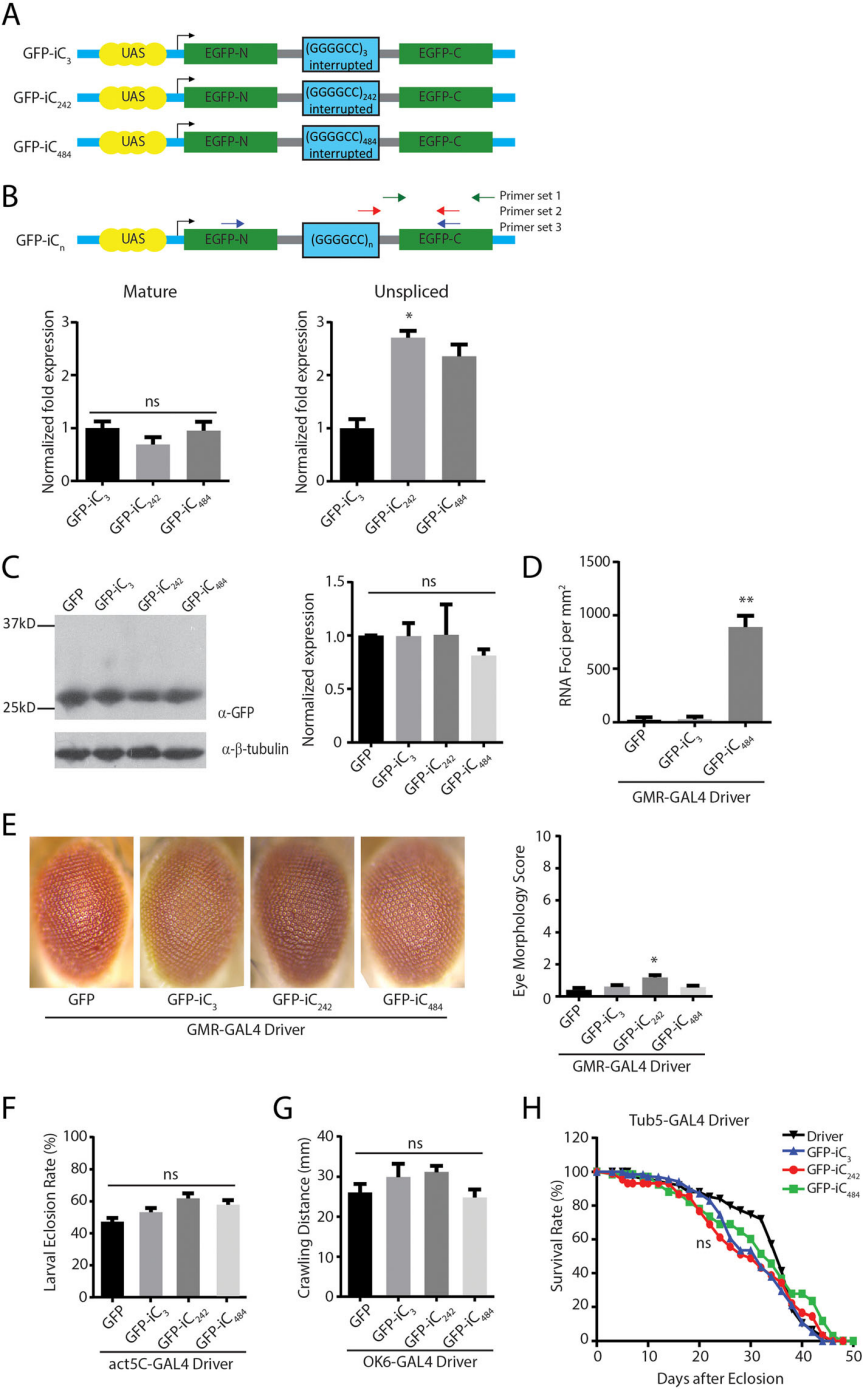
Intronic G4C2 repeats influence splicing and elicit RNA foci in *Drosophila.***a** Schematic of constructs used to generate transgenic fly lines. The *Prospero* fly intron 1 (gray lines) was introduced into UAS-GFP (blue lines). Either three G4C2 repeats or serial (G4C2)21–28 repeat units separated by 14 nt interruptions were serially inserted into the intron. **b** Locations of the primer pairs used for measuring unspliced, spliced, and total mRNA are show in the schematic. Quantification of the expression of the mature (left) and unspliced (right) GFP product in the indicated fly lines (right), *n* = 9. **c** Western blot of lysates from heads of G4C2 intronic repeat flies (left) with quantification of GFP normalized to beta-tubulin (right), *n* = 5. **d** Quantification of RNA foci in the retina of the indicated intronic fly, *n* = 10. **e** Representative eye phenotypes from flies of the indicated genotypes crossed to GMR-GAL4 to drive expression in developing ommatidia (left) and quantification of eye phenotype scores (right). **f** Quantification of the number of progeny carrying the transgene after flies of the indicated genotypes were crossed to a ubiquitous driver (act5c-GAL4). **g** Quantification of the distance crawled by 3rd instar larvae from the crosses of the indicated fly genotypes to a larval motor neuron specific driver (OK6-GAL4). **h** Flies carrying the indicated transgenes were crossed to a Tubulin Geneswitch driver (Tub5) to allow ubiquitous expression post eclosion. Adult male flies were placed on food containing RU-486 to activate gene expression and their viability was tracked over 50 days. Graphs represent means ± SEM. * *p* < 0.05; ** *p* < 0.01 by Kruskal-Wallis after Dunn’s correction for multiple comparisons. The number of flies for each genotype for E, F, G and H was > 100

To create an intronic repeat model of C9ALS/FTD, we inserted the first intron of the fly *Prospero* gene [32] into the sequence of GFP in a fashion that retained the canonical splice site donor and acceptor sequences (Fig. 1a). We then inserted either three G_4_C_2_ repeats or concatamers of 28 and 21 G_4_C_2_ repeat units with short interruptions into the intron, up to a maximal repeat length of 484 repeats (Supplemental Figure S1, Supplemental Table S1). After initial studies demonstrated no significant phenotype associated with 28 or 49 intronic repeats (not shown), we focused our analysis on lines expressing 3 (iC_3_), 242 (iC_242_), or 484 (iC_484_) intronic repeats.

The G_4_C_2_ repeat as RNA can form a strong G-quadruplex secondary structure that might interfere with RNA metabolism and splicing [18, 46, 47]. To determine if the repeat taken out of its native context is capable of eliciting alterations in mRNA splicing, we measured the total, unspliced, and spliced mRNA from fly lines containing 3, 242, or 484 intronic repeats (Fig. 1b). There was no significant difference in the abundance of spliced *GFP* RNA across repeat sizes (Fig. 1b). Consistent with this, the amount of GFP protein expressed in the intronic repeat flies was comparable to that seen in flies expressing GFP alone and did not decline with larger repeat sizes (Fig. 1c). In contrast, there was a significant repeat-length dependent increase in unspliced pre-mRNA containing the G_4_C_2_ repeat when normalized to *RPL32* (Fig. 1b). Additionally, RNA foci were observed in flies expressing 484 G_4_C_2_ repeats but not 3 repeats (Fig. 1d and Supplemental Figure S3).

We utilized the UAS-GAL4 system to target expression of the intronic repeats to different tissues. Expression of GFP-iC_3_, GFP-iC_242_, or GFP-iC_484_ in the eye using a GMR-GAL4 driver elicited modest toxicity in GFP-iC_242_ eye phenotypes compared to a control line expressing GFP alone using a standardized toxicity scale [35] (Fig. 1e). No significant eye phenotypes were observed in GFP-iC_484_ expressing lines. Similarly, activating ubiquitous expression during development using an Actin5C-GAL4 driver elicited no differences in eclosion rates between expanded intron lines and the control GFP line (Fig. 1f). We also selectively activated expression of the intronic repeat transgenes in motor neurons using an OK6-GAL4 driver line and evaluated larval crawling as a motor phenotype. We observed no difference between short and long intronic repeats compared to flies expressing GFP alone (Fig. 1g). To evaluate whether a reduction in lifespan might be elicited in adulthood, we activated transgene expression in adult male flies after eclosion using a Geneswitch Tubulin-GAL4 driver by addition of RU-486 to the fly food. We observed no significant impact of intronic repeat expression on viability (Fig. 1h).

### The role of repeat location in GGGGCC toxicity

**Fig. 2:**
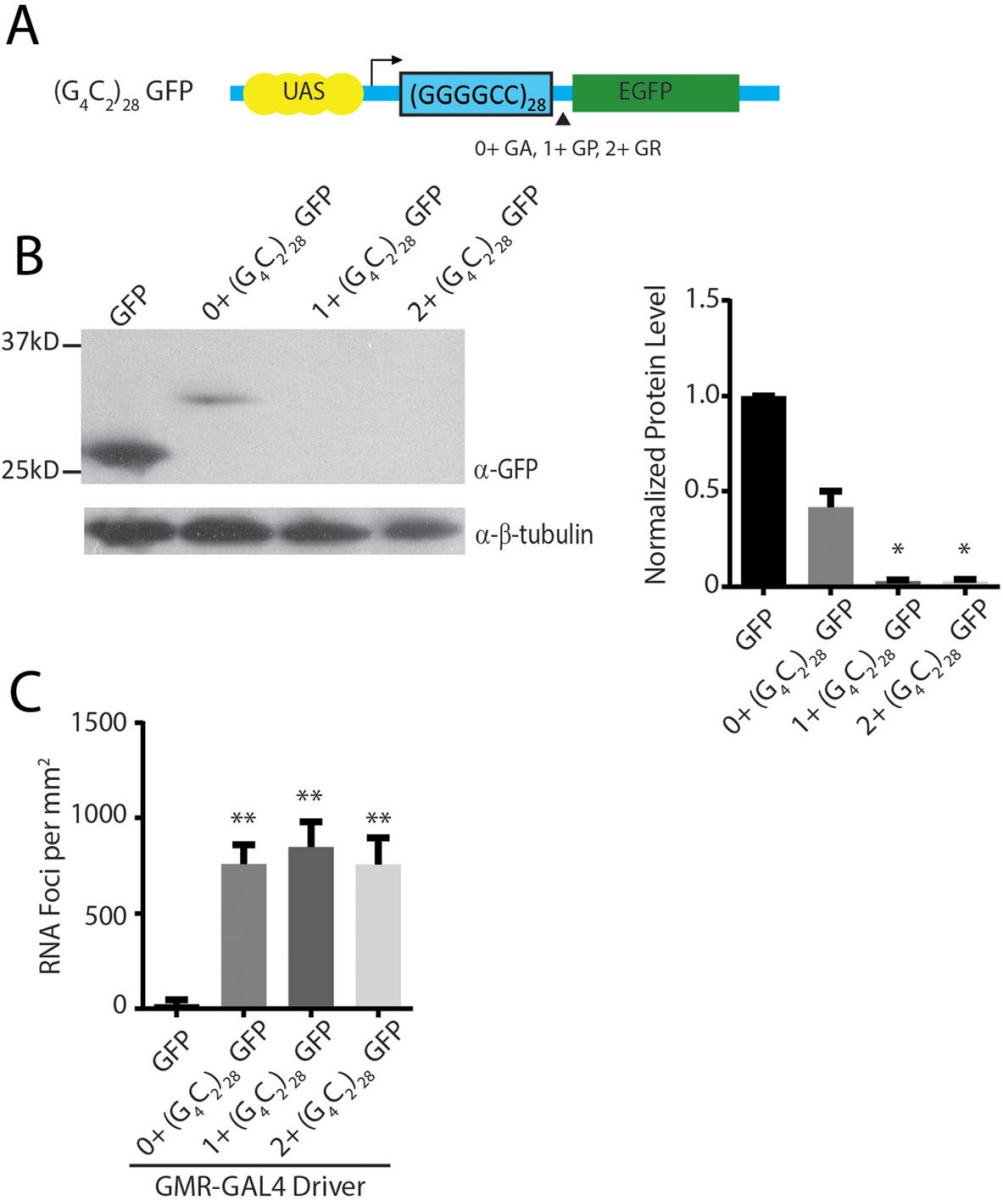
*Drosophila* 5’UTR G4C2 repeat models. **a** Schematic of constructs used to generate transgenic fly lines. (G4C2)28 repeats were inserted into the 5’UTR of GFP without an upstream start codon in all three reading frames relative to the GFP reading frame. **b** Western blot (left) of lysates from heads of G4C2 exonic repeat flies normalized to β-tubulin (right), *n* = 6. **c** Quantification of RNA foci in the retina of the indicated fly lines, n = 10. Graphs represent means ± SEM. * p < 0.05; ** *p* < 0.01 by Kruskal-Wallis after Dunn’s correction for multiple comparisons

To determine if the repeat location within a transcript might impact its toxicity, we inserted the same 28 G_4_C_2_ repeat cassette used to generate the intronic repeats into the 5′ leader of GFP rather than an intron (Fig. 2a). No start codons were present between the transcription start site and the repeat, although the ATG of GFP was retained 3′ to the repeat. We introduced small frameshift mutations between the repeat and the open reading frame of GFP so that it acted as a reporter for RAN translation in the 0+ (GA), 1+ (GP), or 2+ (GR) reading frames, respectively.

When the G_4_C_2_ repeat was expressed in an intronic context, levels of GFP protein were similar between short repeats and expanded repeats. However, placing the repeat in the 5′ leader of GFP dramatically affected overall GFP expression. A higher molecular weight GFP fusion protein product was observed by western blot in the 0+ (GA) frame, but undetectable in the GP (1+) or GR (2+) reading frames (Fig. 2b), though the impact of such RAN translation DPRs in GFP protein solubility was not fully explored. Expression of all three constructs led to detectable RNA foci (Fig. 2c and Supplemental Figure S3). The presence and abundance of RNA foci did not directly predict toxicity in these flies, similar to prior observations [28].

**Fig. 3:**
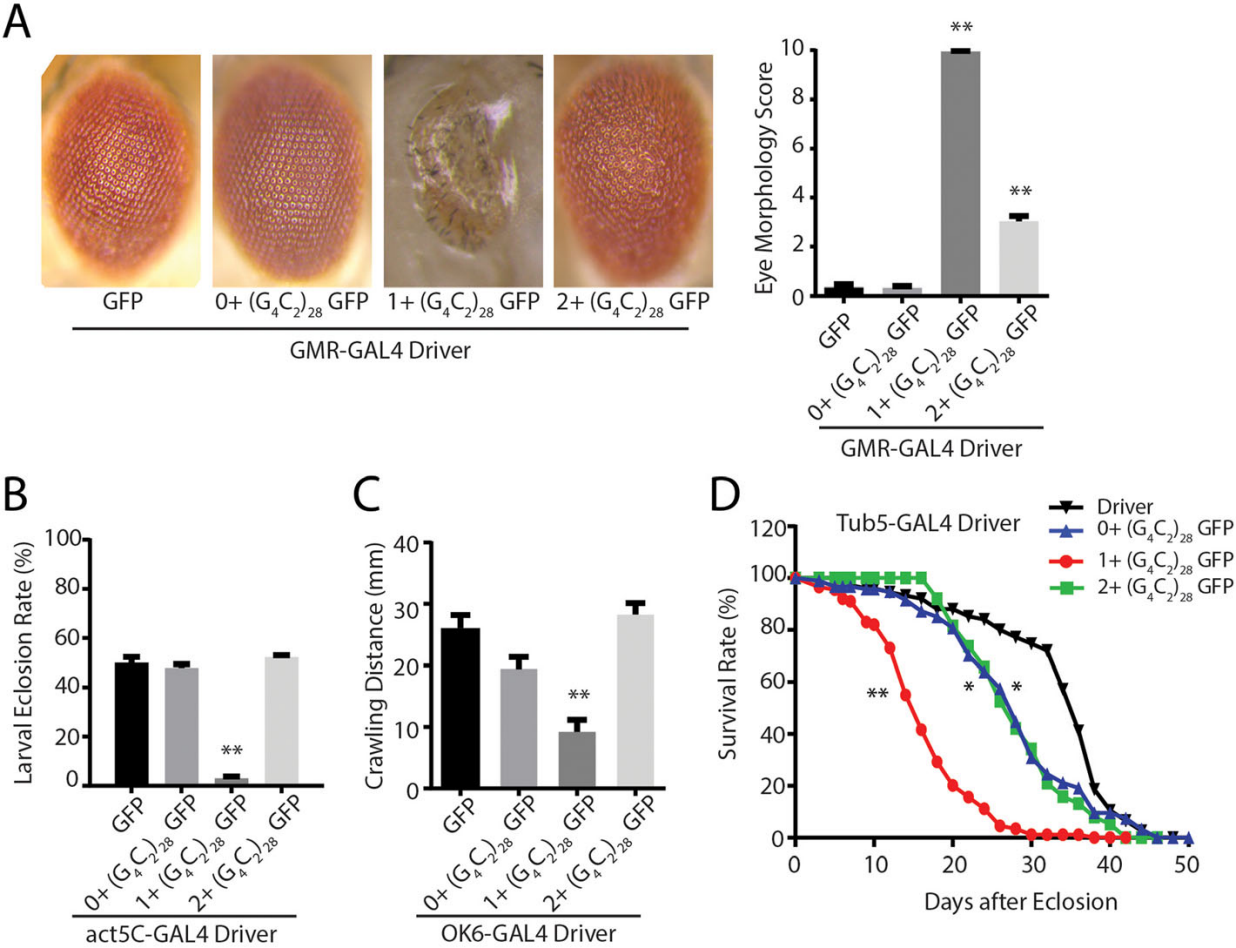
5’UTR G4C2 repeats with different carboxyl termini elicit differential toxicity in *Drosophila***a** Representative eye phenotypes from flies of the indicated genotypes crossed to GMR-GAL4 to drive expression in developing ommatidia (right) and quantification of eye phenotype scores (right). **b** The number of progeny carrying the transgene was determined after flies of the indicated genotypes were crossed to a ubiquitous driver (act5c-GAL4). **c** Quantification of the distance crawled by 3rd instar larvae from the crosses of the indicated fly genotypes to a larval motor neuron specific driver (OK6-GAL4). **d** Flies carrying the indicated transgenes were crossed to a Tubulin Geneswitch driver (Tub5) to allow ubiquitous expression post eclosion. Adult male flies were placed on food containing RU-486 to activate gene expression and their viability was tracked over 50 days. Graphs represent means ± SEM. ** *p* < 0.01 by Kruskal-Wallis after Dunn’s correction for multiple comparisons. Log-rank (Mantel-Cox) test for survival, * *p* < 0.05, ** *p* < 0.01. The number of flies for each genotype was > 100

When expressed in fly ommatidia, 5′ leader repeat fly lines elicited greater toxicity than was observed in the intronic repeat fly lines across a range of measures, consistent with a prior publication [28]. However, surprisingly, there were significant differences in the toxicity observed that was dependent on the reading frame in which the GFP reporter was placed. Specifically, 1+ (G_4_C_2_)_28_ GFP fly lines elicited a marked degeneration of the eye (Fig. 3a) and to a lesser extent in 2+ (G_4_C_2_)_28_ GFP flies. Developmental toxicity, as assessed by eclosion rates after ubiquitous expression, and a severe larval motor phenotype observed with isolated motor neuron expression, largely mirrored toxicity findings observed in the eye (Fig. 3b-c). Moreover, rapid declines in viability after transgene activation in adulthood were observed in all 5′ leader flies, with the most robust toxicity in 1+ (G_4_C_2_)_28_ GFP fly lines (Fig. 3d).

In an attempt to explain these differences in phenotype, we compared transgene mRNA expression across lines and observed no correlation between toxicity and abundance of the transgene expression (Supplemental Figure S1B). We next analyzed three independent lines for each transgene to control for effects of transgene insertion sites. Phenotypes for different lines of the same transgene were very similar despite significant differences across transgenes (Supplemental Figure S1C). Of note, the DNA sequence 5′ to the repeat in all three transgene constructs were identical, precluding contributions from an upstream initiation event in only one construct as a driving force in the observed phenotypic differences (Supplemental Table S1).

### Accumulation and localization of DPR protein correlates with toxicity

To determine what factors might be triggering the differential toxicity between these *Drosophila* lines, we used a series of new and recently developed [40] antibodies against the three sense strand DPRs: GA, GR, and GP. These polyclonal antibodies recognize epitope tagged versions of the indicated repeat proteins both by western blot and by immunocytochemistry in transfected cells (Supplemental Figure S2A-B). These antibodies also stain perinuclear inclusions in cerebellar slices from ALS patients with *C9ORF72* repeat expansions but not in healthy controls (Supplemental Figure S2C).

**Fig. 4:**
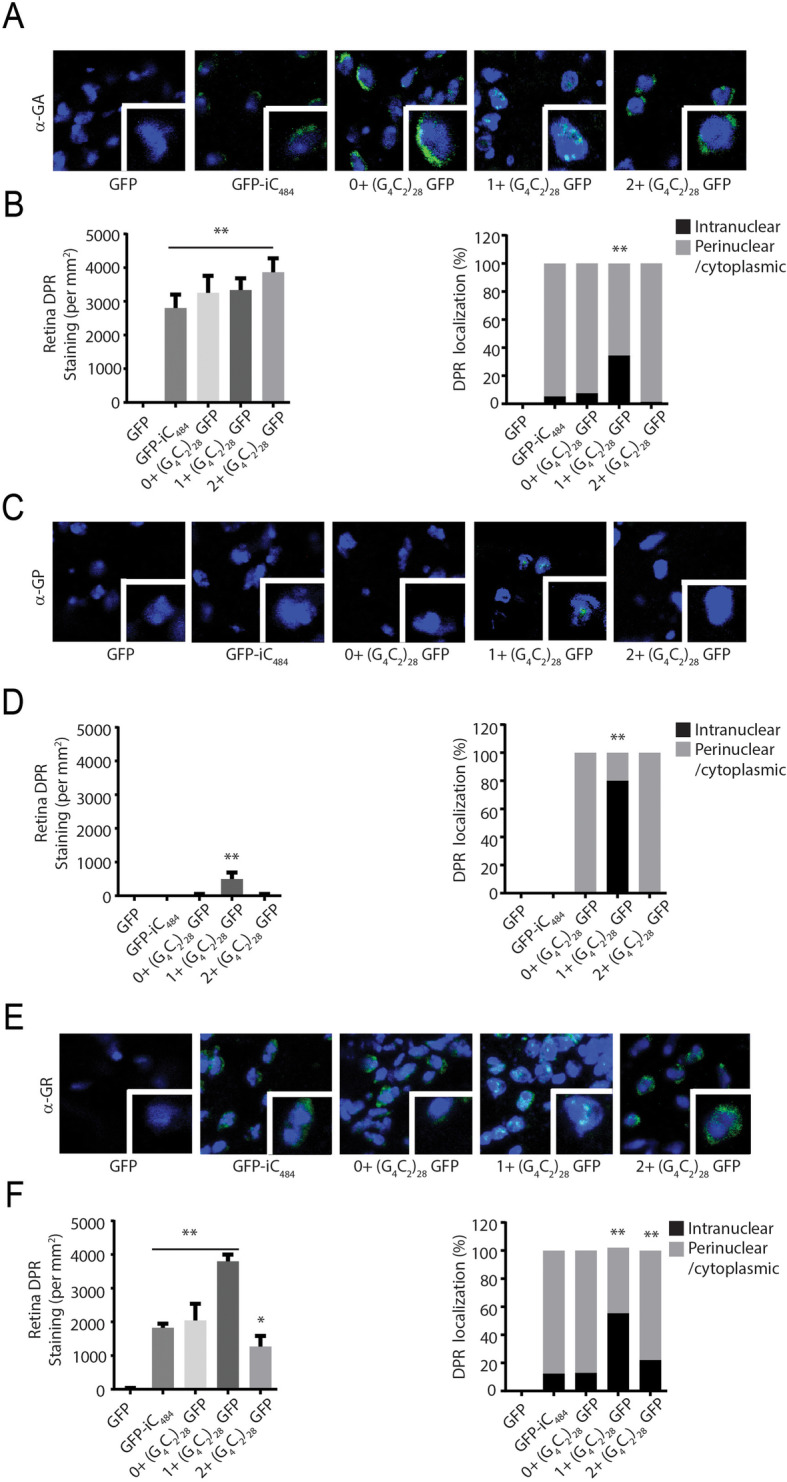
G4C2 repeats toxicity is associated with nuclear accumulation of RAN translation peptides. **a** Representative images of transverse retinal sections from *Drosophila* of the indicated genotypes probed with antibody against GA dipeptide repeats. **b** Quantification of total GA repeat staining by immunofluorescence (left) and the percentile of cells with significant nuclear accumulation of GA DPR staining for the indicated genotypes (right). **c** Representative Immunofluorescence images of *Drosophila* retinal sections probed with antibodies against the GP dipeptide repeats. **d** Quantification of total GP repeat staining by immunofluorescence (left) and the percentile of cells with significant nuclear accumulation of GP DPR staining for the indicated genotypes (right). E) Representative Immunofluorescence image of transverse retinal sections from *Drosophila* of the indicated genotypes probed with antibody against GR dipeptide repeats. F) Quantification of total GR repeat staining by immunofluorescence (left) and the percentile of cells with significant nuclear accumulation of GR DPR staining for the indicated genotypes. Graphs represent means ± SEM. * p < 0.05; ** p < 0.01 by Kruskal-Wallis after Dunn’s correction for multiple comparisons. The number of flies analyzed for each respective genotype in B, D and F was 10

We utilized these antibodies to determine the relative abundance and distribution of each DPR in all of the transgenic *Drosophila* lines described. We reasoned that the epitope tags provide information on a single reading frame, but that RAN translation occurs in all 3 reading frames and thus the relative abundance of each product might be informative for their roles in toxicity. The expected RAN translation products from the intronic constructs would produce 3 possible polypeptides with mixed all three DPRs in one frame, and pure poly (GP)_28_ in the second frame, and mixed poly (GR) and poly (GA) DPRs in the third frame (Table S1), while the expected RAN translation products from all three 5′ leader constructs would just produce 3 pure individual DPRs in each respective reading frames (Table 1). Using transverse ommatidial sections derived from *Drosophila* expressing each transgene (UAS-GFP, GFP-iC_3_, GFP-iC_484_, and 0+, 1+, or 2+ (G_4_C_2_)_28_ GFP lines) crossed to GMR-GAL4, we performed immunofluorescence analysis against each of the three DPRs using these new antibodies. No staining was observed in lines expressing GFP in isolation or in the driver line alone. However, staining was consistently present with both GA and GR antibodies in the intronic fly models (Fig. 4). In contrast, GP staining was not reliably observed from the intronic fly lines. In 5′ leader lines there were marked differences in the staining for both GR and GP between lines, with the greatest staining present in the 1+ (G_4_C_2_)_28_ GFP lines which also exhibited the greatest toxicity (Fig. 4c-dD). In addition, this same line exhibited a marked increase in both total nuclear staining and in the nuclear-cytoplasmic ratio for all three DPR proteins (Fig. 4d), suggesting a correlation between both abundance and cellular localization of these proteins and the observed toxicity in *Drosophila* [20, 25, 28, 48].

**Table 1:** RAN Translation Peptides in G4C2 repeat transgenic flies and plasmids

Genotype	In (GA) n frame	In (GP) n frame	In (GR) n frame
C9ORF72 sense strand	(GA) _n_ Cterminal 31 AAs	(GP) _n_ Cterminal 34 AAs	(GR) _n_ Cterminal 54 AAs
	(GA) _n_WSGRARGRARG GAAVAVPAPAAAEAQ AVASG*	(GP)_n_GRGRGGPGGGPGAGLRLRCLRPRRRRRRRWRVGE*	(GR)_n_GVVGAGPGAGPGRGCGCGACARGGGGAGGGEWVSEEAASWRVAVWGSAAGKRRG*
0+ (G_4_C_2_)_28_ GFP	(GA)_28_ Cterminal253AAs(GFP)	(GP)_28_ Cterminal 15 AAs	(GR)_28_ Cterminal 258 AAs
	(GA)_28_GAWSGRARDP PVAT-GFP*	*(GP)_28_GRGRGGPGIHRSPPW*	(GR)_28_GVVGAGPGST... AAYNHYK*
1+ (G_4_C_2_)_28_ GFP	(GA)_28_ Cterminal 264 AAs	(GP)_28_ Cterminal 258 AAs (GFP)	(GR)_28_ Cterminal 20 AAs
	(GA)_28_GAWSGRARDP... KRPRL*	*(GP)_28_GRGRGGPGIHRS DLEPVAT-GFP*	(GR)_28_GVVGAGPGSTGQISNRSPPW*
2+ (G_4_C_2_)_28_ GFP	(GA)_28_ Cterminal 21 AAs	(GP)_28_ Cterminal 264 AAs	(GR)_28_ Cterminal 258 AAs (GFP)
	(GA)_28_GAWSGRARDPPVRSRNRSPPW*	*(GP)_28_GRGRGGPGIH.... KRPRL*	(GR)_28_GVVGAGPGSTGQISKPVAT-GFP*
ATG-V5 GA/GP/GR CT	(GA)_71_ Cterminal 39 AAs	(GP)_71_ Cterminal 40 AAs	(GR)_71_ Cterminal 60 AAs
	(GA)_71_GRGRVGAPWSGRARGRARGGAAVAVPAPAAAEAQAVASG*	(GP)_71_VVEGWARRGRGGPGGGPGAGLRLRCLRPRRRRRRRWRVGE*	(GR)_71_SWKGGRAVVGAGPGAGPGRGCGCGACARGGGGAGGGEWVSEEAASWRVAVWGSAAGKRRG*
ATG-V5 GA/GP/GR Δ CT	(GA)_71_ Cterminal 9 AAs	(GP)_71_ Cterminal 10 AAs	(GR)_71_ Cterminal 7 AAs
	(GA)_71_GRGRVGAPE*	(GP)_71_VVEGWARLSE*	(GR)_71_ SWKGGRA*

Prior studies have demonstrated that C-terminal sequences and epitope tags can influence the toxicity associated with translated repeat expansions such as polyglutamine proteins [30]. More recently, the C-terminus of the RAN translated protein FMRpolyG generated from GGC repeats in FXTAS was found to influence the relative toxicity of this protein through aberrant protein-protein interactions [31]. We therefore analyzed the C-termini of the transgenes used to generate the different exonic flies (Table 1). In the native *C9ORF72* gene, each DPR reading frame has 30–55 amino acids after the repeat prior to the presence of a stop codon. Such C-termini were detectable in ALS patients carrying the expanded G_4_C_2_ repeats according to a previous study [13]. In our exonic flies, 2 reading frames have C-termini over 200 amino acids, one of which is the fused GFP, while the other is a long polypeptide with no known homology with human proteins, whereas the third reading frame is much shorter (15–21 amino acids).

**Fig. 5:**
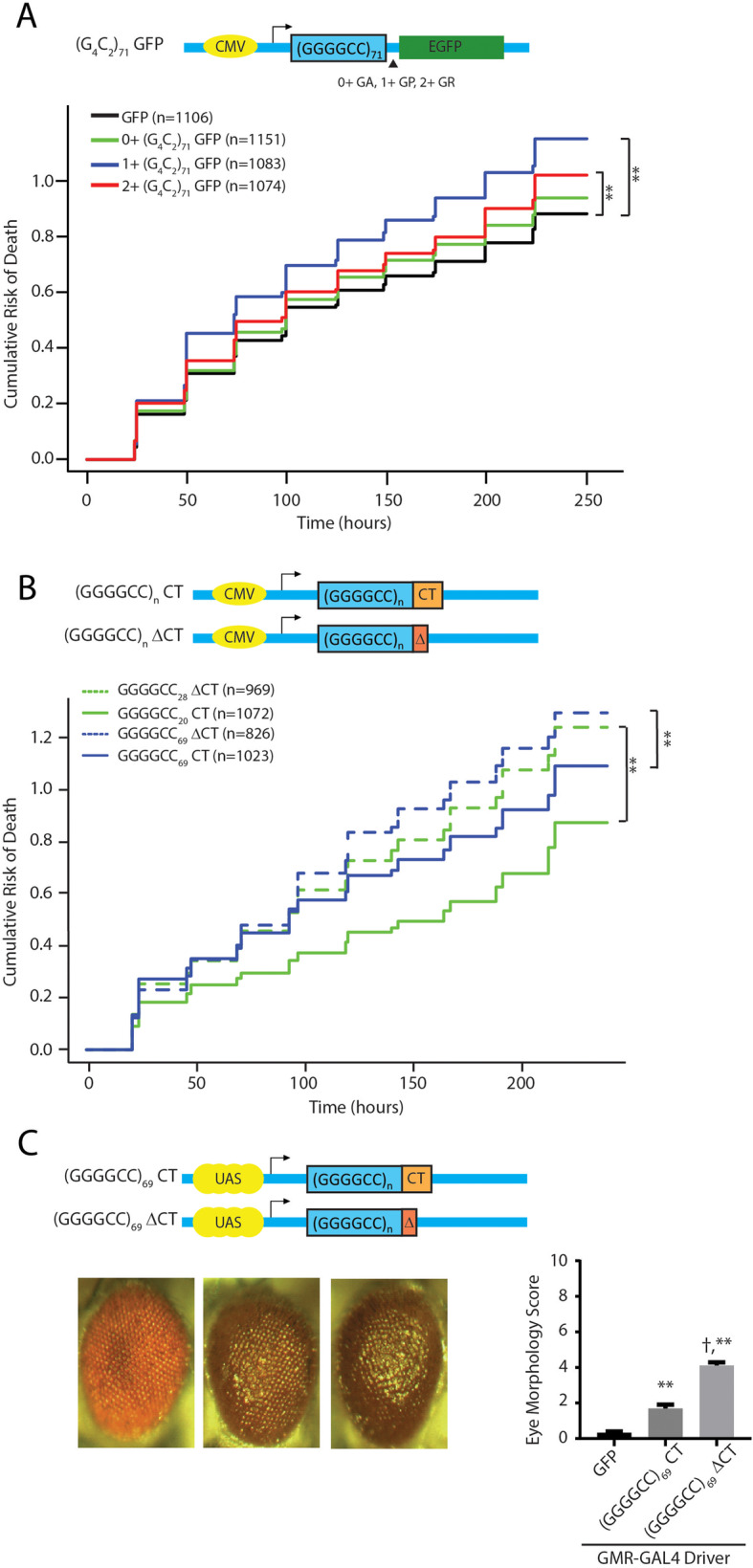
Native carboxyl terminal sequences reduce GGGGCC repeat toxicity in rodent neurons and in flies. **a** Top: Schematic of the constructs transfected into neurons, with GFP placed in different reading frames relative to the GGGGCC repeat. Bottom: Neuronal survival of neurons expressing these constructs is graphed as the cumulative risk of death (Y axis, with higher values reflecting increased death) over time (X-axis, measured in hours). **b** Top: Schematic of the constructs transfected into neurons with shorter or expanded G4C2 repeats and with or without the native Cterminus. Bottom: Cumulative risk of death of neurons. **c** Top: Schematic of the constructs used to generate transgenic fly lines. Bottom: Representative eye phenotypes from flies of the indicated genotypes crossed to GMR-GAL4 to drive expression in developing ommatidia (left) and quantification of eye phenotype scores (right). Graphs represent means ± SEM. For Panels A and B, n = number of neurons. **p < 0.01 difference in survival measured by Cox proportional hazard analysis. Each graph represents at least 3 independent experiments. For panel C, ** p < 0.01 by Kruskal-Wallis after Dunn’s correction for multiple comparisons compared to control group. † p < 0.01 by Kruskal-Wallis after Dunn’s correction for multiple comparisons compared to (G4C2)69 CT group. The number of flies analyzed in panel C was > 100

Based on these observations, we hypothesized that the differential C-termini alone or combined with the DPRs could alter the stability and or distribution of such DPRs and subsequently alter the toxicity. To explore this idea further, we utilized longitudinal microscopy [31, 43–45] to track survival of individual neurons expressing sequences similar to those used in the fly experiments (Fig. 5a). Primary rodent cortical neurons were transfected with plasmids encoding mApple, to visualize neuronal soma, and each of the 5′ leader G_4_C_2_ constructs. These cells were imaged at 24 h intervals over a 10d period, and the time of death for individual neurons determined programmatically using a set of sensitive criteria (rounding or blebbing of the cell body or neurites, loss of fluorescence) validated in previous studies [49, 50]. Relative survival in each condition was compared by Cox proportional hazards analysis. As we observed in *Drosophila,* expression of an expanded G_4_C_2_ repeat in the 5′ UTR of GFP was toxic in neurons. The relative toxicity of each construct was again dictated by the reading frame of the GFP tag, with toxicity being greatest in those with 1+ (G_4_C_2_)_71_GFP or 2+ (G_4_C_2_)_71_ GFP, mirroring what was observed in *Drosophila* (Fig. 5a).

To evaluate the potential role of different C-terminal sequences on toxicity, we generated a new series of expression vectors that take into account the length and sequence of the C-termini. One set of vectors have the native C-termini (CT) for all three reading frames. Another set of vectors was generated with shortened C-terminal regions in all three reading frames (ΔCT) (Fig. 5b). The G_4_C_2_ repeat with the native C-terminal sequence showed increased toxicity with an increasing repeat size (Fig. 5b, solid green vs solid blue line). However, when the native C-termini were removed and replaced with short C-termini, toxicity was significantly increased (Fig. 5b, dashed lines vs solid lines). These (G_4_C_2_)_69_ repeats with the native C-termini (CT) or with short C-termini (ΔCT) were also introduced to the same locus (AttP40) in *Drosophila* using pUAST-AttB vectors to achieve equal expression levels. Consistent to the partially protective effects found in the culture neurons, the inclusion of the native C-termini partially suppressed the repeat-associated toxicity when these constructs were expressed in the fly eyes (Fig. 5c).

**Fig. 6:**
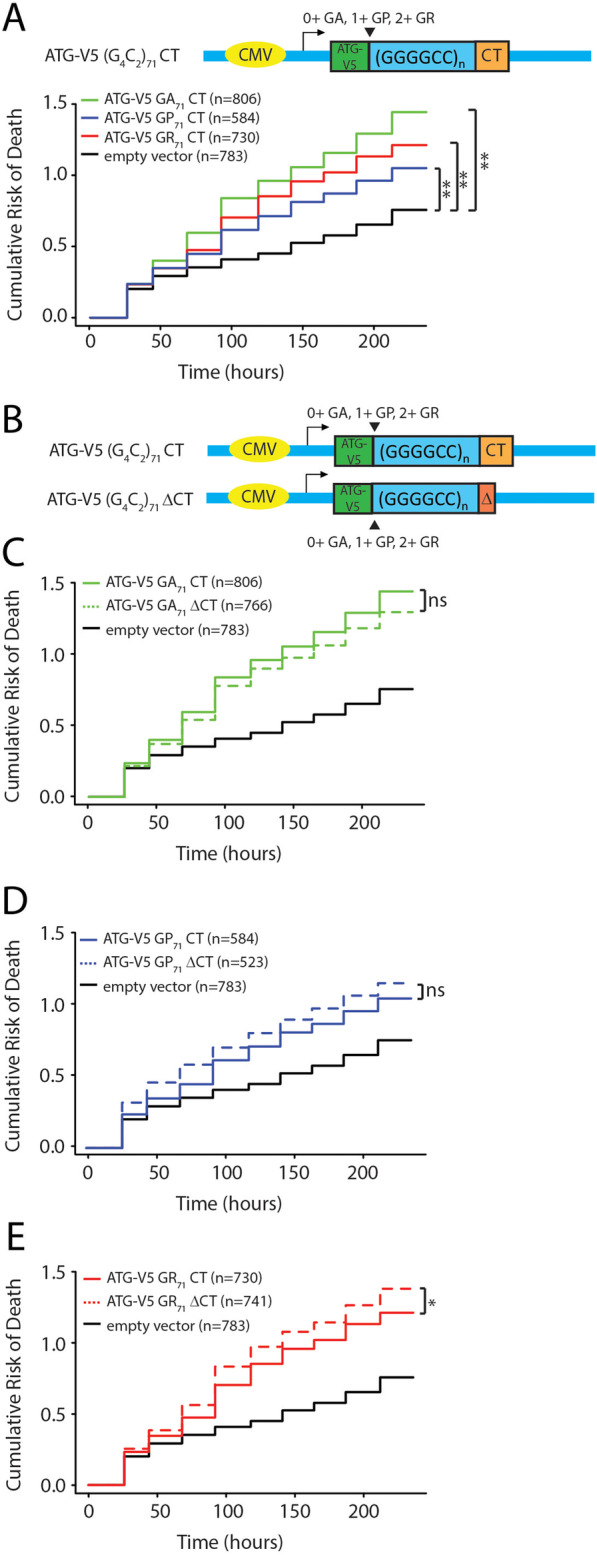
Native carboxyl terminal sequences decrease GR dipeptide repeat licited toxicity in rodent neurons. **a** Top: Schematic of constructs generated to evaluate impact of including an AUG codon in each individual reading frame of the repeat while maintaining the native C-terminal sequence across all 3 reading frames. Bottom: Survival of neurons expressing the indicated constructs as measured by cumulative risk of death. **b** Schematic of constructs used in panels **c**-**e**. **c** Cumulative risk of death of neurons transfected with constructs containing an AUG codon in the GA reading frame with or without the native C-terminus. **d**) Cumulative risk of death of neurons transfected with constructs containing an AUG codon in the GP reading frame with or without the native C-terminus. **e** Cumulative risk of death of neurons transfected with constructs containing an AUG codon in the GR reading frame with or without the native C-terminus. *p < 0.05 and ** p < 0.01 indicate a difference in survival as determined by Cox proportional hazard analysis. The number of neurons for each analysis were as indicated in the respective lines. Each graph represents at least 3 independent experiments

In an attempt to determine which C-terminal reading frame was most critical for toxicity, we introduced a series of AUG driven V5 tags upstream of the repeat in different reading frames. Enhancing production of GA or GR from these repeats significantly boosted the toxicity of these constructs to a greater degree than placement of an AUG codon in the GP reading frame. However, replacing the native C-terminal sequences with stop codons only enhanced repeat toxicity when the AUG codon was placed in the GR reading frame (Fig. 6c-e). Taken together, these data suggest that the native C-termini may mitigate toxicity arising from RAN translation, and in particular toxicity related to GR DPR proteins.

## Discussion

How hexanucleotide repeat expansions in *C9ORF72* elicit neurodegeneration is a topic of intense research [51, 52]. Work by multiple groups has established the presence of both nuclear RNA foci and RAN translation derived DPR proteins in tissues and cells from affected patients. Initial studies largely relied on expression of the repeat out of its normal sequence context or production of DPR proteins via AUG-initiated translation. These studies importantly demonstrate that even relatively short RNA repeats and small DPRs can be toxic [19, 20, 22–26, 53–55]. The position of the GGGGCC repeat within an mRNA is a major factor in determining its relative toxicity in *Drosophila,* with repeats residing in an intron exhibiting minimal toxicity while even short repeats in the 5′ leader elicit significant toxicity [28]. In this work we observe that sequences 3′ to the repeat also modulate repeat toxicity by altering the C-termini of DPRs generated via RAN translation. These findings suggest a specific role for C-terminal sequences in modulating repeat toxicity in C9ALS/FTD while making it clear that repeat sequence context needs to be carefully considered in future studies of disease pathogenesis.

Sequences located in coding regions or within the 5′ or 3′ UTR are typically processed to capped and polyadenylated mRNAs and rapidly trafficked out of the nucleus, where they interface with a series of RNA binding proteins and the translational machinery. In contrast, intronic sequences are typically spliced out during transcription, formed into RNA lariats and targeted for debranching and degradation in the nucleus. As such, one potential explanation for the observed differences in toxicity between intronic and 5′ leader sequence repeats is the distribution of the repeat containing transcripts within the cell and their potential for translation. In vitro and cell based studies using reporter systems suggest that while GGGGCC repeats can be translated from intronic or bi-cistronic transcripts, their production is most robust when present in capped and poly-adenylated mRNAs [56–60]. Consistent with this prediction, the abundance of specific RAN translation products from relatively short 5′ leader repeats were much greater than that observed from larger intronic repeats (Figs. 1, 2, 3 and 4). These differences were also consistent with past studies of DPRs production in in vitro, cellular and *Drosophila* models of GGGGCC repeats [21, 28, 57]. Moreover, the relative abundance and distribution of these DPR proteins was predictive of toxicity, while the total RNA abundance and RNA foci counts were similar across repeat containing lines, consistent with prior studies [21, 28]. While studies in mammalian or human model systems at endogenous expression levels are needed to fully arbitrate the relative roles of RNA and protein in GGGGCC repeat toxicity, our work is consistent with the emerging consensus supporting a significant role of RAN translation in C9ALS/FTD pathogenesis.

In contrast, our finding that the frame in which the C-terminal epitope tag resides was strongly predictive of repeat toxicity is surprising. This finding was quite robust, as we observed this same relationship across multiple different *Drosophila* lines with distinct insertion sites (Supplemental Figure S1) and also when these same constructs were expressed in rodent neurons (Fig. 6). The “GFP frame” where the toxicity was greatest would create a fusion with a GP dipeptide repeat protein—the least toxic DPR based on multiple studies using AUG driven expression. Thus, it could be that “out of frame” untagged products are largely driving toxicity while the GFP tag is actually suppressing toxicity derived from certain DPRs. In this context, it is intriguing that the small sequence changes we introduced below the repeat to shift the reading frame resulted in a short disordered 20 amino acid C-terminal tail in the GR reading frame and a long polypeptide with no known homology along the GA DPR reading frame from the 1+ (G_4_C_2_)_28_ GFP flies with the most severe phenotypes. Our data suggests that these C-terminal changes correlated with both more aggregates of GR DPR in 1+ (G_4_C_2_)_28_ GFP flies and greater nuclear localization of all three DPRs. Moreover, modulating C-terminal sequences in transcripts expressed in rodent neurons had the greatest effect on AUG-driven GR associated toxicity (Fig. 6). However, poly (GA) DPRs can interact with poly (GR) DPRs to contribute the repeat toxicity [25] and are themselves toxic in isolation in mammalian neurons [22, 23, 61] - a finding that is consistent with our own data (Fig. 6a). Thus, while our data is most consistent with a primary role of GR in modulating this C-terminal toxicity, it does not preclude important roles for GA or GP in this process. Future studies will be needed to determine how C-terminal changes to these DPRs in isolation might impact their interactions and toxicity.

Isolated expression of GR DPRs in the absence of the RNA repeat exhibit marked toxicity in *Drosophila,* rodent and cell based model systems [20, 25, 53, 62, 63]. As such, changes in its relative solubility, stability or distribution may well influence the toxicity elicited by G_4_C_2_ repeats. In contrast, the relative lack of toxicity associated with the 0+ (G_4_C_2_)_28_ GFP transgene may also be partially explained by a stabilizing effect of the GFP tag on the GA DPR. Isolated expression of GA DPRs is toxic in mammalian neurons and, to a lesser extent, in *Drosophila* [20, 22, 23, 61]. However, this GA-GFP fusion product remains largely cytoplasmic and soluble in our assays, which may impede GR mediated toxicity in *Drosophila* [25]. Regardless, our results suggest that the C-terminal region of RAN derived proteins influences their relative toxicity and cellular distribution: a finding with significant implications for design of future model systems at both this and other disease causing repeat expansions.

In rodent neurons, we observed a protective effect when the native C-termini are retained in all three reading frames. Based on studies where we serially enhanced expression of each DPR reading frame by inserting an AUG codon in a strong Kozak context above the GGGGCC repeat element, this protective effect seems to be largely driven by the GR reading frame, as elimination of the C-terminus in GR driven constructs enhanced toxicity while doing so in GA or GP driven constructs had no significant or even a mildly protective effect. Given the propensity for GR DPRs to phase separate [64–66], this native C-terminus may influence the behavior of these peptides when generated in patients. Future studies will be needed to determine if and how such surrounding sequences, especially the downstream sequences influence the expression and localization of these DPRs.

It is noteworthy that in rodent neurons with the protective effects of the native C-termini on the expanded GGGGCC repeat toxicity with AUG were not as prominent as that on the repeats without AUG (Figs. 5b vs 6). Several potential mechanisms might explain this discrepancy: First, since the DPR production is tightly tied to toxicity in C9orf72 overexpression models, it may be that the toxic effects of the ATG-driven DPRs overwhelm the subtler contribution from the C-termini. Second, because the ATG-driven constructs also contain a V5 tag, they have a larger N-terminal extension on the DPR that might also influence the impact of C-terminal sequences. Third, RAN translation may be more permissive of initiation in multiple frames, allowing for production of all 3 DPR products within a given neuron rather than one major reading frame, and in this context the C-termini may have larger impacts. A full array of characterizations of these DPRs and their interactions with/without the native C-termini will be needed to dissect these mechanisms in future studies.

In summary, our findings demonstrate that the position of the repeat within a transcript as well as its immediate surrounding sequences can have a major impact on its relative toxicity, and on the distribution and abundance of different RAN translated proteins. Future work will be needed to determine the selective effects that these sequence modifications have on the biophysical properties of RAN translated proteins and their exact roles in disease pathogenesis in ALS and FTD. When coupled with recent findings in FXTAS [31] and Spinocerebellar Ataxia type 36 [67, 68], these findings suggest that similar analyses in other repeat disorders are likely warranted.

## Supplementary information

**Additional file 1: Supplemental Table S1.** Sequences for G_4_C_2_ Repeat-containing pUAST Vectors. The detailed DNA sequences for generating GFP-tagged G_4_C_2_ repeat–containing vectors.

**Additional file 2: Supplemental Table S2.** Primers used for cloning, in situ, and qPCR. The detailed primer sequences used in the various experimental settings.

**Additional file 3: Supplemental Figure S1.** Repeat size, RNA expression, and eye phenotypes for intronic and exonic G_4_C_2_ repeat flies. The characterization of multiple lines of transgenic flies

**Additional file 4: Supplemental Figure S2.** Characterization of dipeptide repeat antibodies. The antibodies targeting the dipeptide repeats effectively detected the respective antigen in transfected cells and in patient cells.

**Additional file 5 Supplemental Figure S3.** Characterization of G_4_C_2_ repeat RNA foci. The Cy5 labeled 2′-O-Me-(CCCCGG)_5_ RNA probe detected the RNA foci in long repeat-containing intronic and 5′-leader repeat flies.

## Data Availability

Not applicable.

## Abbreviations

ALS: Amyotrophic lateral sclerosis
FTD: Frontotemporal dementia
RAN translation: Repeat-associated non-AUG initiated translation
DPR: Dipeptide repeats
UTR: Untranslated region
FXTAS: Fragile X-associated tremor/ataxia syndrome
GA: Glycine-alanine
GP: Glycine-proline
GR: Glycine-arginine
C-terminal: Carboxyl terminal
