# Supplementary material for: The carboxyl termini of RAN translated GGGGCC nucleotide repeat expansions modulate toxicity in models of ALS/FTD
Source: Acta Neuropathol Commun. 2020 Aug 4;8:122. doi: 10.1186/s40478-020-01002-8 (PMC7401224; doi:10.1186/s40478-020-01002-8)
Supplement: Supplementary file 1 — Additional file 1: Supplemental Table S1. Sequences for G4C2 Repeat-containing pUAST Vectors. The detailed DNA sequences for generating GFP-tagged G4C2 repeat–containing vectors. [file 40478_2020_1002_MOESM1_ESM.pdf]

Supplemental Table S1: Sequences for Intronic G4C2 Repeat-containing pUAST Vectors

Name	Sequence	Comments
GFP-intron	GAATTCGCCACCATGGTGAGCAAGGGCGAGGAGCTGTTCACCGGGGTGGTGCCC<br>ATCCTGGTCGAGCTGGACGGCGACGTAAACGGCCACAAGTTCAGCGTGTCCGGC<br>GAGGGCGAGGGCGATGCCACCTACGGCAAGCTGACCCTGAAGTTCATCTGCACC<br>ACCGGCAAGCTGCCCGTGCCCTGGCCCACCCTCGTGACCACCCTGACCTACGGC<br>GTGCAGTGCTTCAGCCGCTACCCCGACCACATGAAGCAGCACGACTTCTTCAAGT<br>CCGCCATGCCCGAAGGCTACGTCCAGGAGCGCACCATCTTCTTCAAGGACGACG<br>GCAACTACAAGACCCGCGCCGAGGTGAAGTTCGAGGGCGACACCCTGGTGAACC<br>GCATCGAGCTGAAGGgtgagtttccacctagtccaccgtgtgcagcagaaacgagacagagagagagaga<br>gagagagagacggggagaaaagtagaagtagtagtagtggtagaaaggaa**gcggccgcctcgag**ggcgcgcc<br>actagtgctagcggtaccccttagatctatctataccccctctctctccctctgtggctctgccccacacggacatggaaatttg<br>gaaagacaatcgaccatccatccgacacacatatctcattcatataccctatatctataacgccaccagGCATCGAC<br>TTCAAGGAGGACGGCAACATCCTGGGGCACAAGCTGGAGTACAACTACAACAGC<br>CACAACGTCTATATCATGGCCGACAAGCAGAAGAACGGCATCAAGGTGAACTTCA<br>AGATCCGCCACAACATCGAGGACGGCAGCGTGCAGCTCGCCGACCACTACCAGC<br>AGAACACCCCCATCGGCGACGGCCCCGTGCTGCTGCCCGACAACCACTACCTGA<br>GCACCCAGTCCGCCCTGAGCAAAGACCCCAACGAGAAGCGCGATCACATGGTCC<br>TGCTGGAGTTCGTGACCGCCGCCGGGATCACTCTCGGCATGGACGAGCTGTACA<br>AGTAATCTAGA	Backbone for all GFP-intronic constructs. Upper cases for exons while lowercase for artificial introns. Green for interrupted GFP gene, and red for the intronic MCS, with individual restriction sites NotI and XhoI underlined in bold.
NotI-(GGGGCC)$_{3\ \text{or}\ 21}$-XhoI	GCGGCCGCTACGCATCCCAGTTTGAGACGGGGGCCGGGGCCGGGGCCGGGGC<br>GTGGTCGGGGCGGGCCCGGGGGCGGGCCCGGGGCGGGGCTGCGGTTGCGGT<br>GCCTGCGCCCGCGGCGGCGGAGGCGCAGGCGGTGGCGAGTGGGTGAGTGAGG<br>AGGCGGCATCCTGGCGGGTGGCTGTTTGGGGTTCGGCTGCCGGGAAGAGGCGC<br>GGGTAGAAGCGGGGGCTCTCCTCAGAGCTCGACGCATTTTTACTTTCCCTCTCAT<br>TTCTCTGACCGAAGCTGGGTGTCGGGCTTTCGCCTCTAGCGACTGGTGCTCGAG	Insert for short GGGGCC repeats, with repeat region makes as yellow.
NotI-(GGGGCC)$_{28}$-XhoI	GCGGCCGCCCGCAGCCTGTAGCAAGCTCTGGAACTCAGGAGTCGCGCGCTAGG<br>GGCCGGGGCCGGGGCCGGGGCCGGGGCCGGGGCCGGGGCCGGGGCCGGGG<br>CCGGGGCCGGGGCCGGGGCCGGGGCCGGGGCCGGGGCCGGGGCCGGGGCCG<br>GGGCCGGGGCCGGGGCCGGGGCCGGGGCCGGGGCCGGGGCCGGGGCCGGG<br>GCCGGGGCCGGGGCCGGGGCGTGGTCGGGGCGGGCCCGGGGGCGGGCCCGG<br>GGCGGGGCTGCGGTTGCGGTGCCTGCGCCCGCGGCGGCGGAGGCGCAGGCGG<br>TGGCGAGTGGGTGAGTGAGGAGGCGGCATCCTGGCGGGTGGCTGTTTGGGGTT<br>CGGCTGCCGGGAAGAGGCGCGGGTAGAAGCGGGGGCTCTCCTCAGAGCTCGAC<br>GCATTTTTACTTTCCCTCTCATTTCTCTGACCGAAGCTGGGTGTCGGGCTTTCGCC<br>TCTAGCGACTGGTGCTCGAG	Insert with 28 GGGGCC repeats marked in yellow. Note the PspOMI site in red for the future self- insertion to make C242 and C484.
NotI-(GGGGCC)$_{21}$-PspOMI	GCGGCCGCTACGCATCCCAGTTTGAGACGGGGGCCGGGGCCGGGGCCGGGGC<br>CGGGGCCGGGGCCGGGGCCGGGGCCGGGGCCGGGGCCGGGGCCGGGGCCG<br>GGGCCGGGGCCGGGGCCGGGGCCGGGGCCGGGGCCGGGGCCGGGGCCGGG<br>GCCGGGGCGTGGTCGGGCCC	Building block of 21 GGGGCC repeats to generate C49, C70, C91 and C121.
Concatamerized repeat sequences in C242 and C484	GC[GGCCGCCCGCAGCCTGTAGCAAGCTCTGGAACTCAGGAGTCGCGCGCTAGG<br>GGCCGGGGCCGGGGCCGGGGCCGGGGCCGGGGCCGGGGCCGGGGCCGGGG<br>CCGGGGCCGGGGCCGGGGCCGGGGCCGGGGCCGGGGCCGGGGCCGGGGCCG<br>GGGCCGGGGCCGGGGCCGGGGCCGGGGCCGGGGCCGGGGCCGGGGCCGGG<br>GCCGGGGCCGGGGCCGGGGCGTGGTCGGGGCGGGCC(GCTACGCATCCCAGTT<br>TGAGACGGGGGCCGGGGCCGGGGCCGGGGCCGGGGCCGGGGCCGGGGCCGG<br>GGCCGGGGCCGGGGCCGGGGCCGGGGCCGGGGCCGGGGCCGGGGCCGGGG<br>CCGGGGCCGGGGCCGGGGCCGGGGCCGGGGCCGGGGCGTGGTCGGGCC)$_4$]$_{2\ \text{or}}$<br>$_4$	Potential RAN translation from repeats in both iC_{242} and iC_{484}<br>Frame 1:<br>(GA)$_{28}$GAWSGRAATHPSLRR[(GP)$_{21}$GRGRAATHPSLRR]$_3$(GP)$_{21}$GRGRAGRPQPVASSGTQESRAR(GR)$_{28}$GVVGAGRYASQFET[(GA)$_{21}$GAWSGRYASQFET]3(GA)$_{21}$WSGRPPAACSKLWNSGVAR*<br>Frame 2: (GP)$_{28}$GRGRGGPLRIPV*<br>Frame 3:<br>(GR)$_{28}$GVVGAGRYASQFET[(GA)$_{21}$GAWSGRYASQFET]$_3$(GA)$_{21}$WSGRPPAACSKLWNSGVAR*
