# Supplementary material for: The carboxyl termini of RAN translated GGGGCC nucleotide repeat expansions modulate toxicity in models of ALS/FTD
Source: Acta Neuropathol Commun. 2020 Aug 4;8:122. doi: 10.1186/s40478-020-01002-8 (PMC7401224; doi:10.1186/s40478-020-01002-8)
Supplement: Supplementary file 2 — Additional file 2: Supplemental Table S2. Primers used for cloning, in situ, and qPCR. The detailed primer sequences used in the various experimental settings. [file 40478_2020_1002_MOESM2_ESM.pdf]

Supplemental Table S2: Primers used for cloning, In Situ, and qPCR

For C9ORF72 Intronic repeat cloning

Primer name	Primer sequence
NotI-C9 AnchorR	5’-CCAGCGGCCGCTACGCATCCAGTTTGAGACGGGGGCC-3’
XhoI-C9 F	5’-CCCTCGAGCACCAGTCGCTAGAGGCGAAAGCCCG-3’
NotI-C9R	5’-CCAGCGGCCGCCCGCAGCCTGTAGCAAGC-3’
PspOMI-tiling	5’-AAAGGGCCCGACCACGCCCCGGCC-3’

For G_4C_2 exonic repeat cloning

Primer name	Primer sequence
Adapter1	5’-ACCGGTCAGATCTCGAACCGGT-3’
Adapter2	5’-ACCGGTCAGATCTCGAAACCGGT-3’

For In situ analysis

Primer name	Primer sequence
Probe	5’-/5Cy5N/mGmGmCmCmCrCrGrGrCrCrCrCrGr GrCrCrCrCrGrGrCrCrCrCrGmGmCmCmCmC-3’

For qRT-PCR of GFP in flies

GFP unspliced forward	5’-ACTAGTGCTAGCGGTACCCCTTAGATC-3’
GFP spliced forward	5’-TCTTCTTCAAGGACGACGGCAACTAC-3’
GFP reverse (for spliced+unspliced)	5’-GTACTCCAGCTTGTGCCCCAGGATGT-3’
RPL32 forward	5’-GCCCAGCATACAGGCCCAAG-3’
RPL32 reverse	5’-AAGCGGCGACGCACTCTGTT-3’
