# Supplementary material for: The carboxyl termini of RAN translated GGGGCC nucleotide repeat expansions modulate toxicity in models of ALS/FTD
Source: Acta Neuropathol Commun. 2020 Aug 4;8:122. doi: 10.1186/s40478-020-01002-8 (PMC7401224; doi:10.1186/s40478-020-01002-8)
Supplement: Supplementary file 3 — Additional file 3: Supplemental Figure S1. Repeat size, RNA expression, and eye phenotypes for intronic and exonic G4C2 repeat flies. The characterization of multiple lines of transgenic flies [file 40478_2020_1002_MOESM3_ESM.pdf]

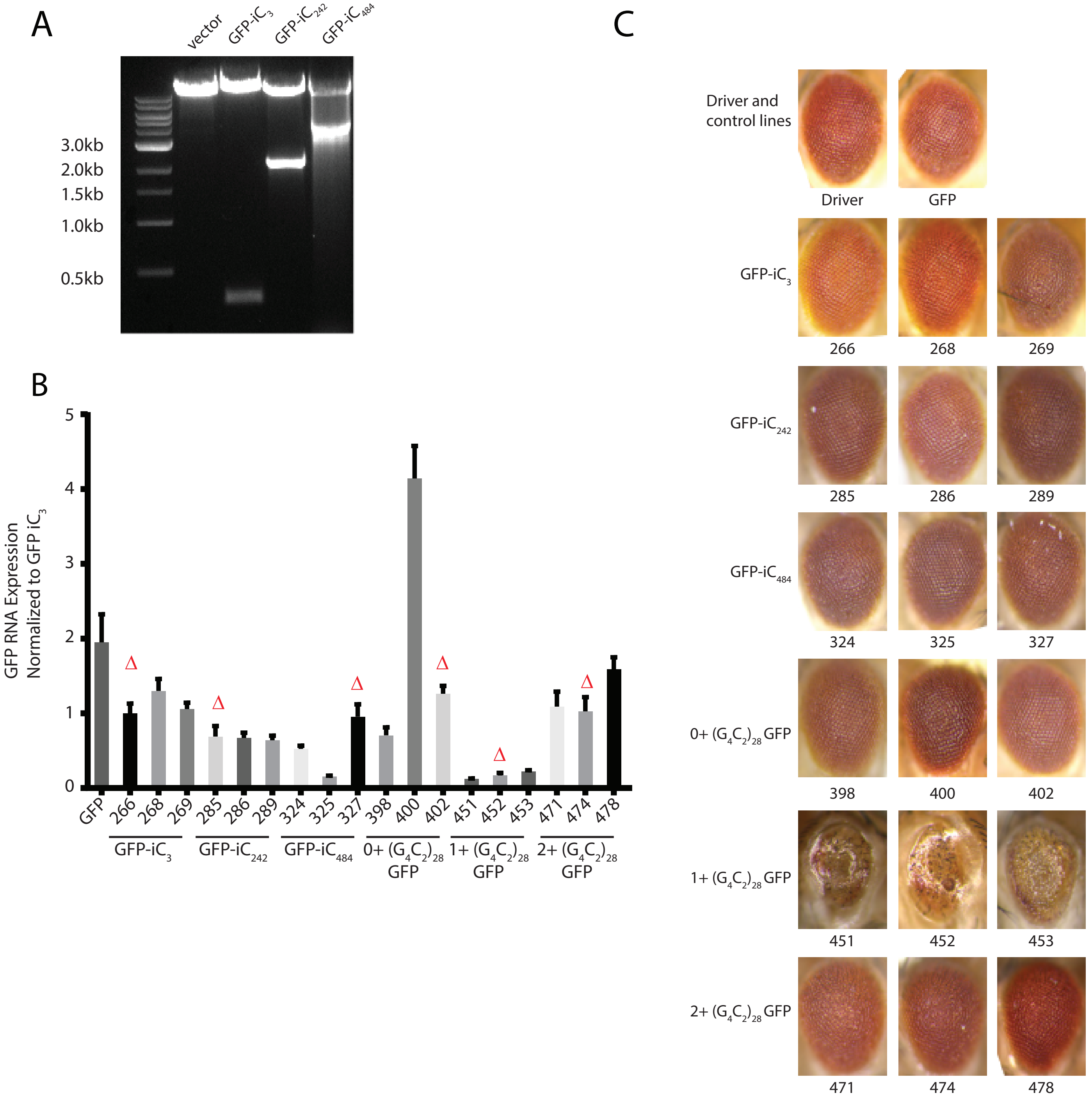
A
vector
GFP-iC3
GFP-iC242
GFP-iC484
3.0kb
2.0kb
1.5kb
1.0kb
0.5kb
B
GFP RNA Expression Normalized to GFP iC3
5
4
3
2
1
0
GFP
266
268
269
285
286
289
324
325
327
398
400
402
451
452
453
471
474
478
GFP-iC3
GFP-iC242
GFP-iC484
0+ (G4C2)28 GFP
1+ (G4C2)28 GFP
2+ (G4C2)28 GFP
C
Driver and control lines
Driver
GFP
GFP-iC3
266
268
269
GFP-iC242
285
286
289
GFP-iC484
324
325
327
0+ (G4C2)28 GFP
398
400
402
1+ (G4C2)28 GFP
451
452
453
2+ (G4C2)28 GFP
471
474
478

**Supplemental Figure S1: Repeat size, RNA expression, and eye phenotypes for intronic and exonic $G_4C_2$ repeat flies.** A) DNA agarose gel validating repeat size of intronic repeat containing vectors. B) GFP mRNA expression from transgenic fly lines. Lines with comparable GFP mRNA levels (Δ) were used for major assays conducted. C) Representative eye images from multiple independent lines for each genotype.
