# Supplementary material for: The carboxyl termini of RAN translated GGGGCC nucleotide repeat expansions modulate toxicity in models of ALS/FTD
Source: Acta Neuropathol Commun. 2020 Aug 4;8:122. doi: 10.1186/s40478-020-01002-8 (PMC7401224; doi:10.1186/s40478-020-01002-8)
Supplement: Supplementary file 4 — Additional file 4: Supplemental Figure S2. Characterization of dipeptide repeat antibodies. The antibodies targeting the dipeptide repeats effectively detected the respective antigen in transfected cells and in patient cells. [file 40478_2020_1002_MOESM4_ESM.pdf]

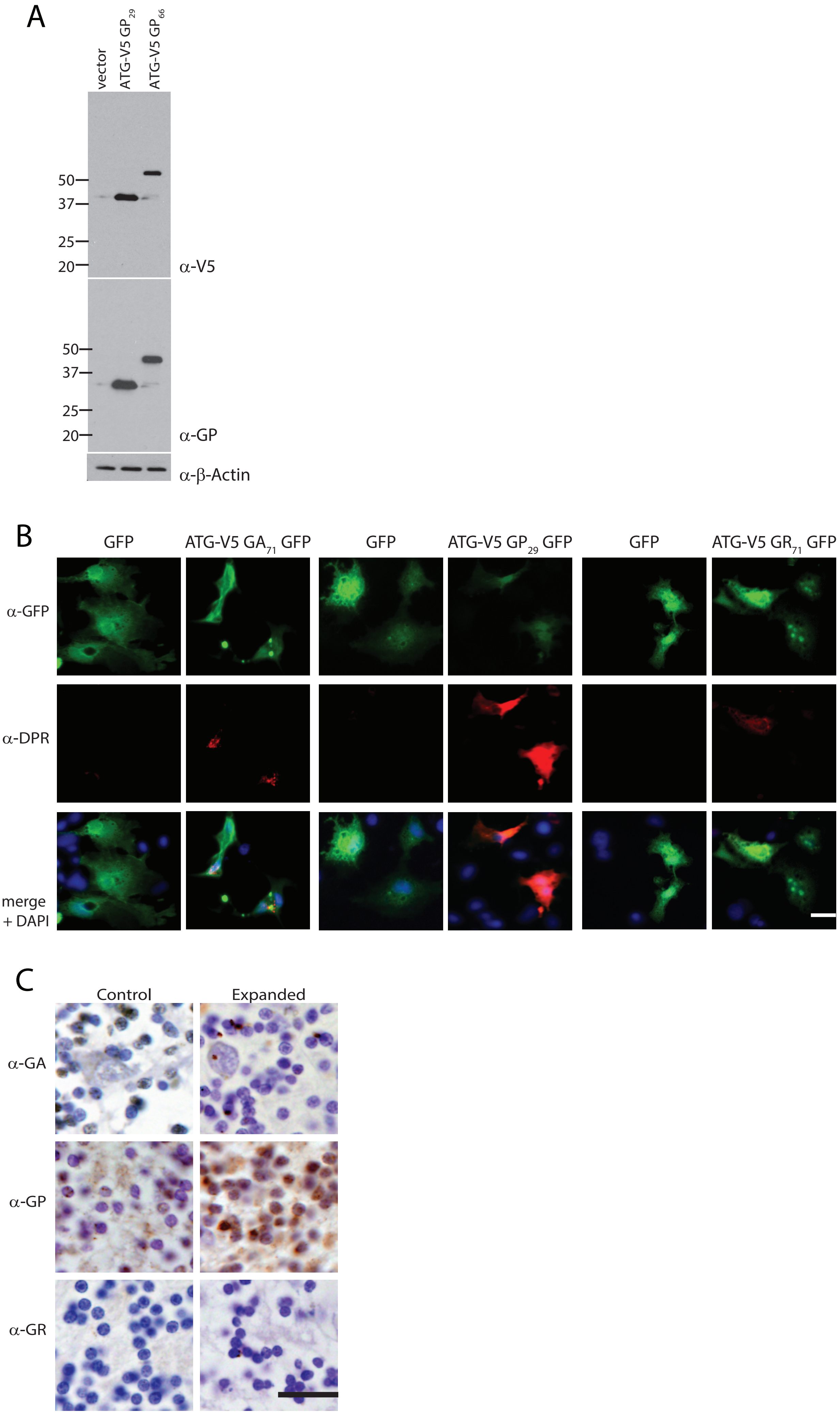
A
vector
ATG-V5 GP29
ATG-V5 GP66
50
37
25
20
α-V5
50
37
25
20
α-GP
α-β-Actin
B
GFP
ATG-V5 GA71 GFP
GFP
ATG-V5 GP29 GFP
GFP
ATG-V5 GR71 GFP
α-GFP
α-DPR
merge
+ DAPI
C
Control
Expanded
α-GA
α-GP
α-GR

Supplemental Figure S2: Characterization of dipeptide repeat antibodies.
Dipeptide repeat (DPR) antibodies specifically recognize G_4C_2 containing plasmids by western blot (A) and immunofluorescence (B). DPR positive aggregates are visible in patient cerebellar tissue by immunocytochemistry (C). Scale bar of B and C = 20μm.
