# Supplementary material for: The carboxyl termini of RAN translated GGGGCC nucleotide repeat expansions modulate toxicity in models of ALS/FTD
Source: Acta Neuropathol Commun. 2020 Aug 4;8:122. doi: 10.1186/s40478-020-01002-8 (PMC7401224; doi:10.1186/s40478-020-01002-8)
Supplement: Supplementary file 5 — Additional file 5 Supplemental Figure S3. Characterization of G4C2 repeat RNA foci. The Cy5 labeled 2′-O-Me-(CCCCGG)5 RNA probe detected the RNA foci in long repeat-containing intronic and 5′-leader repeat flies. [file 40478_2020_1002_MOESM5_ESM.pdf]

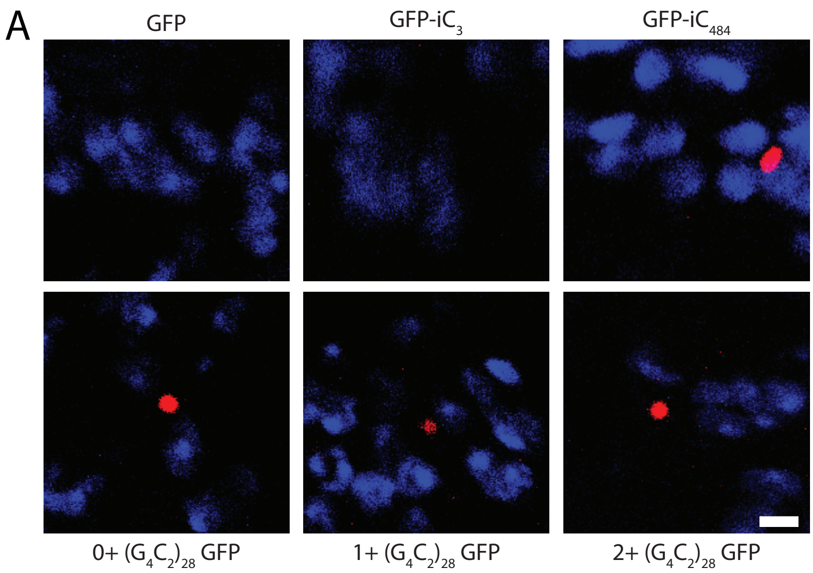
A
GFP
GFP-iC3
GFP-iC484
0+ (G4C2)28 GFP
1+ (G4C2)28 GFP
2+ (G4C2)28 GFP

Supplemental Figure S3: Characterization of G_4C_2 repeat RNA foci in intronic and exonic G_4C_2 repeat flies. Representative images of in situ hybridization using Cy5-$(G_2C_4)_5$ probe in transverse sections of fly ommatidia from respective genotypes crossed to GMR-Gal4 driver. Scale bar = 20μm.
